# Improving sunflower oil stability with propolis: A study on antioxidative effects of Turkish propolis during accelerated oxidation

**DOI:** 10.1111/1750-3841.17482

**Published:** 2024-10-30

**Authors:** Ayhan Baştürk, Berfin Yavaş

**Affiliations:** ^1^ Department of Food Engineering, Faculty of Engineering, Van Yuzuncu Yil University Van Turkey

**Keywords:** ABTS, antioxidant activity, DPPH, methanol extract, natural antioxidant

## Abstract

Propolis, a natural resinous substance collected by bees, is known for its potent antioxidant properties. This study investigates the antioxidant activities and total phenolic contents of propolis samples from 16 provinces of Türkiye and their effects on the oxidative stability of sunflower oil. The 1,1‐diphenyl‐2‐picrylhydrazyl (DPPH) inhibition was in the range of 28.1%–92.5% in thirteen propolis samples, whereas this rate was 24.5% in butylated hydroxytoluene (BHT). Although 2,2′‐azino‐bis(3‐ethylbenzotiazolin‐6‐sulfonic acid) (ABTS) value was 224 µmol trolox/g in BHT, this value was in the range of 262–1370 µmol trolox/g in propolis samples, except for one. Propolis methanol extracts 13 applied to sunflower oil at a concentration of 1000 ppm were more efficient than BHT added at 200 ppm for inhibiting the production of peroxide value (PV). Similarly, most propolis extracts (1000 ppm) demonstrated antioxidant activity against the production of p‐anisidine (p‐AV) in sunflower oil under accelerated oxidation conditions. It was determined that Turkish propolis had strong antioxidant properties and delayed oxidation and may be utilized commercially in the food sector to delay the oxidation of fats and oils.

## 1 INTRODUCTION

Spoilage in fat containing foods primarily occurs due to autooxidation and hydrolysis. These reactions cause quality losses, including bad taste and rancidity, in both crude and refined oils, and lead to the deterioration of their functional and nutritional properties. The stability and degradation of oils depend on their ingredients, processing, and storage conditions. The autooxidation of fats is a process that takes place through free radical chain reactions. This is a catalytic process involving hydroperoxide formation and other oxidation, degradation, and polymerization reactions (Rodriguez‐Amaya & Shahidi, 2021; Schaich, 2023).

Antioxidants are frequently used to improve the oxidative stability of edible oils. They function in different ways depending on which part of the lipid oxidation pathway they interfere with. Antioxidants are generally classified as primary antioxidants, secondary antioxidants, chelators, quenchers, oxygen scavengers, or antioxidant regenerators. Antioxidants are also divided into two groups, synthetic and natural, according to the source from which they are obtained. Synthetic antioxidants are widely used worldwide because of their low cost, ease of production, and high performance in retarding lipid oxidation. For example, butylated hydroxytoluene (BHT) is a synthetic antioxidant widely used to protect oils against thermal and oxidative degradation and to inhibit free radical formation. In recent years, despite the effective role of these chemicals in preventing oxidation of fats and oils, there has been growing concern about their carcinogenic, mutagenic, or, under certain conditions, harmful effects on humans (Indiarto & Qonit, 2020; Xu et al., 2021). This has led to producers and consumers developing natural antioxidants. Therefore, instead of synthetic antioxidants, oils should be enriched with substances containing high amounts of natural antioxidant compounds.

Natural antioxidants are compounds that plants or living food tissues have developed to recover from oxidative stress by controlling free radicals, fat oxidation catalysts, and oxidation primary and secondary breakdown products. These antioxidant compounds include flavonoids, phenolic acids, carotenoids, and tocopherols, which can inhibit oxidation, scavenge free radicals, and act as reducing agents (Akbari et al., 2022; Rahaman et al., 2023; Zufarov & Serkayev, 2024). In addition, some plant essential oils also have significant antioxidant properties. For example, essential oils of some peperina varieties (*Calamintha nepeta*, *Hedeoma multiflorum*, and *Minthostachys verticillata*) have been reported to show significant antioxidant activity (Juncos et al., 2024). In another study, *Origanum vulgare* and *Humulus lupulus* essential oils were reported to increase the oxidative stability of sunflower oil below 150°C (López et al., 2023).

Currently, there is a growing interest in propolis, a product that bees collect from plants and partly add their bodily secretions, for its natural immune‐boosting, antimicrobial, and antioxidant properties. The literature is abundant with studies examining the contents, antioxidant, and antimicrobial properties of propolis extracts (Akman et al., 2023; Altuntaş et al., 2023; Arslan et al., 2021; Can et al., 2023; Degirmencioglu et al., 2019; Kekecoglu et al., 2022; Kolaylı et al., 2023; Maden Çalışkol, 2013; Svecnjak et al., 2020). However, the effects of these extracts on the oxidative stability of oils have not yet been thoroughly investigated. Therefore, it is crucial to explore the potential of propolis extracts in preventing the degradation of oils. Özcan (2000) tested the antioxidant activity of propolis methanol extracts of natural olive oil stored at 60°C. The concentration of the extracts in olive oil ranged from 0.02% to 0.08%. Extracts at concentrations of 0.06% and 0.08% exhibited better antioxidant activity than butylated hydroxy anisole (BHA) and BHT at 0.01% levels. Esfandiarifard and Ziaolhagh (2021) added different concentrations of methanolic propolis extract (0%, 0.5%, and 1%) to sunflower oil, incubated at 25°C for 60 days and investigated its antioxidant activity. The results showed that the addition of antioxidants to sunflower oil led to a decrease in peroxide value (PV), thiobarbituric acid reactive substances (TBARS), and acidity values of the samples. The 1% methanol extract of propolis was more potent than the 0.5% extracts and TBHQ (synthetic antioxidant). Uçak (2018) conducted a study using propolis extract as a natural antioxidant in fish oil. In all of the studies mentioned above propolis extracts showed significant antioxidant effects. Although numerous studies have examined the antioxidant properties of propolis, there is limited research on its effects on the oxidative stability of oils. This study aims to fill this gap by investigating the antioxidant activity of propolis from various regions in Türkiye and its impact on sunflower oil stability.

Sunflower oil is preferred owing to its widespread use. The share of sunflower oil among edible vegetable oils in Türkiye is approximately 70% (Karakaş, 2020). Sunflower oil is widely used as a major dietary source of linoleic acid. Normal sunflower oil is characterized by a high concentration of linoleic acid, followed by oleic acid. The high unsaturated fatty acid content of sunflower oil makes it susceptible to oxidation.

In this study, propolis was collected from 16 provinces in Türkiye (Afyon, Bitlis, Hatay, Kocaeli, Aydın, Balıkesir, Çorum, Ordu, Ankara, Giresun, Kilis, Antalya, Van, Karaman, Adıyaman, and Rize). The antioxidant activity and total phenolic content (TPC) of the propolis samples were determined. Methanol extracts obtained from propolis samples were added to sunflower oil and subjected to accelerated oxidation. The PV, conjugated diene (K_232_), and conjugated triene (K_270_) as primary oxidation products, p‐anisidine (p‐AV) as a secondary oxidation product, total oxidation value (TOTOX), and free fatty acidity (FFA) as an indicator of hydrolytic reactions were determined for these oil samples at certain periods. By comparing these results, the effects of propolis obtained from different regions and flora on the oxidative stability of sunflower oil were determined. The aim of this study was to determine the antioxidant activities and TPC of propolis obtained from 16 different provinces of Türkiye and to investigate the effects of methanol extracts of these propolis on the oxidative stability of sunflower oil under accelerated oxidation conditions (60°C, 28 days).

## 2 MATERIALS AND METHODS

### 2.1 Materials

**FIGURE 1:**
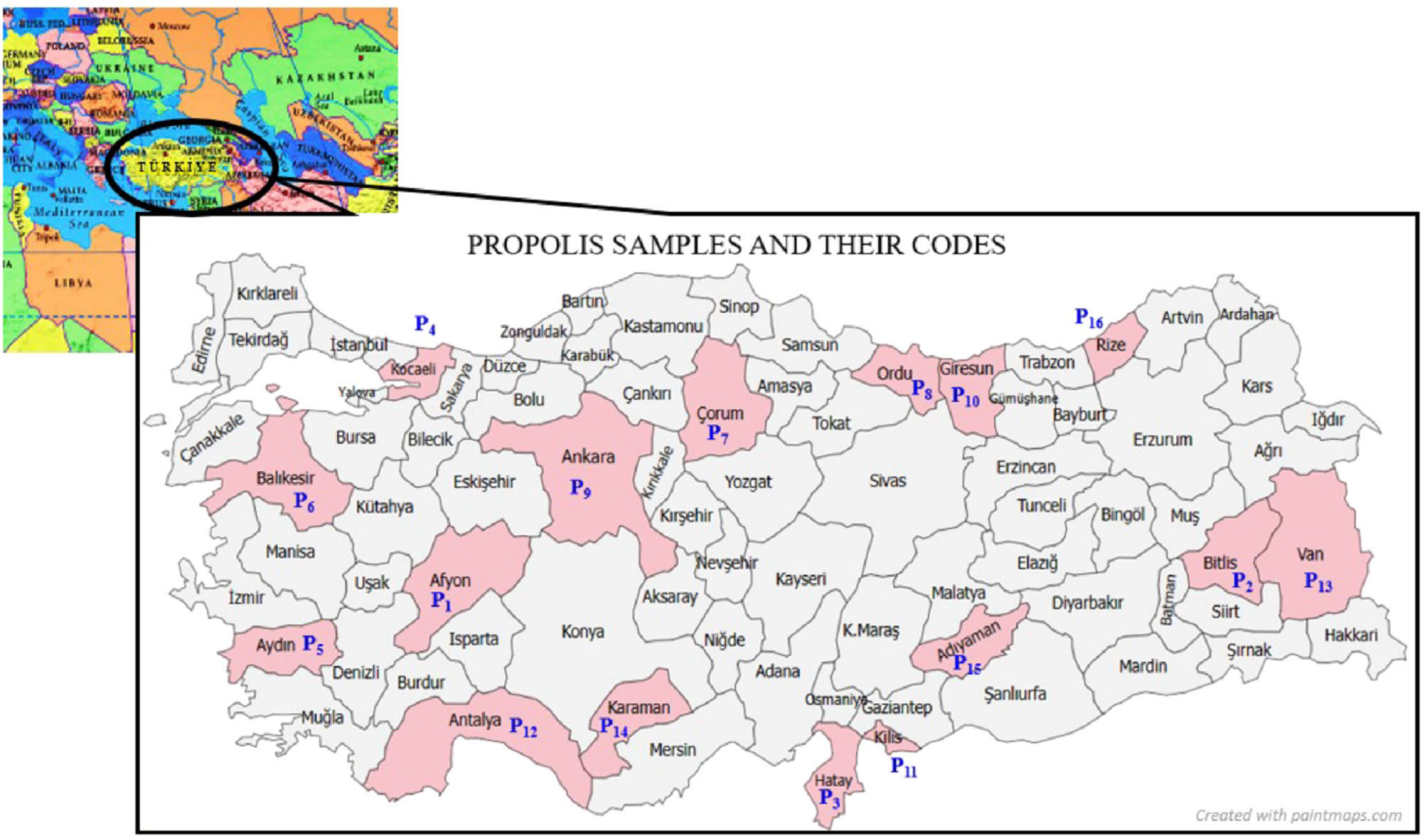
Map of Türkiye highlighting the provinces where propolis samples were collected. Each province is labeled with its corresponding sample code for reference.

Propolis was obtained from 16 regions of Türkiye: Afyon, Bitlis, Hatay, Kocaeli, Aydın, Balıkesir, Çorum, Ordu, Ankara, Giresun, Kilis, Antalya, Van, Karaman, Adıyaman, and Rize from May to October 2019. The provinces where propolis was collected in Türkiye and their codes are shown in Figure 1. The collected propolis was stored at −18°C until use. BHT, Folin–Ciocalteu indicator, 1,1‐diphenyl‐2‐picrylhydrazyl (DPPH), and 2,2′‐azino‐bis(3‐ethylbenzothiazoline‐6‐sulfonic acid) diammonium salt (ABTS) were obtained from Sigma‐Aldrich. All other chemicals used in the analyses were of analytical purity.

### 2.2 Methods

In this study, the DPPH antioxidant activities, trolox equivalent antioxidant capacity (ABTS), and TPC of propolis obtained from 16 provinces in Anatolia were determined. Then, methanol extracts of these propolis were added to pure sunflower oil (SOp) purified from antioxidants by aluminum oxide treatment at a concentration of 1000 ppm. The concentrations of propolis extracts were chosen considering sufficiently effective concentrations of propolis extracts and kept at the same level for a fair comparison among the samples. Refined sunflower oil (SOr) and BHT containing SOp were used as controls. BHT was used as a control because it is the most widely used synthetic antioxidant for protecting oils against thermal and oxidative degradation. Oil samples were subjected to accelerated oxidation at 60°C for 28 days. Oxidation was carried out in 50 mL open‐mouth amber bottles by retaining 30 mL of the oil samples in an oven adjusted to 60°C. PV, K_232_, K_270_, p‐AV, TOTOX, and FFA were analyzed on Days 0, 7, 14, 21, and 28. In addition, the fatty acid composition was determined in the samples taken on the 1st, 14th, and 28th days. The aim of this study was to determine and compare the antioxidant activities of propolis and its effects on the oxidative stability of sunflower oil.

#### 2.2.1 Defatting propolis

Propolis samples taken from the freezer (−18°C) were pulverized and subjected to extraction with n‐hexane in a Soxhlet apparatus. For this purpose, 10 g of powdered propolis was placed on filter paper in the thimble holder of the Soxhlet apparatus and extracted with 150 mL n‐hexane for 4 h to remove oil and waxy substances. Thus, the oil and waxy substances in propolis were removed. The defatted propolis samples were dried and prepared for use as methanol extract.

#### 2.2.2 Preparation of methanol extracts

Propolis powder purified from oil and waxes by the Soxhlet extraction method was weighed to 10 g and homogenized in 50 mL methanol. HCl (5 µL) was added, and the mixture was shaken at 200 rpm on a circular shaker at room temperature for 3 h. The filtrate was then filtered with Whatman filter paper (No. 4), taken into falcon tubes, and centrifuged at 8000 × *g* for 20 min (4°C). After filtering again using Whatman filter paper, the supernatant was transferred to a separate container. After centrifugation, 10 mL methanol was added to the residue remaining at the bottom of the falcon tube, and the same procedure was repeated. The resulting supernatants were combined, and the methanol content was removed using a rotary evaporator at 50°C. Some of the powdered extracts were weighed and diluted with methanol to a volume of 25 mL. The other portion was reserved for the addition of sunflower oil at 1000 ppm.

#### 2.2.3 Stripping sunflower oil of antioxidants by aluminum oxide treatment

To determine the effects of propolis extracts and other antioxidant substances added to sunflower oil samples, tocopherol homologues present in the oils should be removed initially. The procedure was performed according to Yoshida et al. (1992), with some modifications. An amount of 65 g aluminum oxide, activated by heating at 100°C for 8 h and 200°C for 10 h, was placed on a 3 × 45 cm^2^ column. Initially, 65 mL n‐hexane was passed through the column, and then 65 g of refined sunflower oil was mixed with 65 mL n‐hexane and passed through the column under vacuum. The solvent in the oil that passed through the column was removed under vacuum in a rotary evaporator operated at 40°C and 65 rpm.

#### 2.2.4 Preparation and accelerated oxidation of oil samples

Different propolis extracts were added to each antioxidant‐free sunflower oil (SOp) sample at a concentration of 1000 ppm. First, the required amount of extract was dissolved in ethanol with thorough stirring. Subsequently, it was added to sunflower oil heated to 50°C. The mixture was then magnetically agitated for 1 day at 200 rpm using a magnetic stirrer (Hei‐PLATE, Heidolph). SOp, SOr, and SOp samples to which BHT was added at a concentration of 200 ppm were used as the control group. Here, SOr represents refined sunflower oil. SOp represents sunflower oil purified from antioxidants, such as tocopherol and analogs, that may have been added after refining. Sop + BHT represent sunflower oil containing 200 ppm of synthetic antioxidants. The effect of propolis extracts on oxidative stability will be evaluated in comparison with three different controls. The 19 prepared oil samples were subjected to accelerated oxidation by storage at 60°C for 28 days (Basturk et al., 2007; Javidipour et al., 2015). Samples were collected on Days 0, 7, 14, 21, and 28 and stored in sealed bottles, protected from light, and refrigerated at +4°C until analysis. The oil samples were coded as follows: refined sunflower oil: SOr, sunflower oil stripped of antioxidants: SOp, pure sunflower oil with 200 ppm BHT: SOp + BHT, SOp with 1000 ppm P_1_ added: SOp + P_1_, SOp with 1000 ppm P_2_ added: SOp + P_2_, SOp with 1000 ppm P_3_ added: SOp + P_3_, SOp with 1000 ppm P_4_ added: SOp + P_4_, SOp with 1000 ppm P_5_ added: SOp + P_5_, SOp with 1000 ppm P_6_ added: SOp + P_6_, SOp with 1000 ppm P_7_ added: SOp + P_7_, SOp with 1000 ppm P_8_ added: SOp + P_8_, SOp with 1000 ppm P_9_ added: SOp + P_9_, SOp with 1000 ppm P_10_ added: SOp + P_10_, SOp with 1000 ppm P_11_ added: SOp + P_11_, SOp with 1000 ppm P_12_ added: SOp + P_12_, SOp with 1000 ppm P_13_ added: SOp + P_13_, SOp with 1000 ppm P_14_ added: SOp + P_14_, SOp with 1000 ppm P_15_ added: SOp + P_15_, SOp with 1000 ppm P_16_ added: SOp + P_16_.

### 2.3 Analysis of propolis

#### 2.3.1 Determination of DPPH radical scavenging activity

The DPPH free radical scavenging activity assay was performed using the method proposed by Huang et al. (2005) with some modifications. This method is based on the spectrophotometric measurement of the decrease in color as a result of the destruction of the DPPH (2,2‐diphenyl‐1‐picrylhydrazyl) radical, a stable purple compound. DPPH solution was prepared by dissolving 0.025 g DPPH in 1 L methanol, and this stock solution was stored at −20°C until needed. Accordingly, 0.1 mL of propolis methanolic extract was mixed with 3.9 mL of DPPH solution and incubated at room temperature in the dark for 60 min. At the end of the experiment, the absorbance of the sample was measured at 515 nm against the blind sample in a spectrophotometer (Agilent 8453, Agilent Technologies). The blind sample was prepared by adding 3.9 mL of DPPH solution to 0.1 mL of methanol and following the same procedure as for the sample. The inhibition rate of DPPH radicals was calculated using Equation (1). The results are presented as %inhibition:

(1) $$\%\mathrm{Inhibition}=\frac{\left(\mathrm{Ab}s_{\mathrm{blind}}-\mathrm{Ab}s_{\mathrm{sample}}\right)}{\mathrm{Ab}s_{\mathrm{blind}}}\times 100$$

#### 2.3.2 ABTS^•+^ scavenging ability

The ABTS^•+^ scavenging ability assay was performed using the method previously described by Re et al. (1999) with some modifications. This method provides a relative measurement of the amount of antioxidant substances retained by the ABTS^•+^ radical cation compared to standard amounts of a synthetic antioxidant, trolox (a water‐soluble vitamin E analog). Measurements were made by spectrophotometric determination of the disappearance of the ABTS^•+^ radical, a stable blue/green compound. The reaction between ABTS and potassium persulfate forms a blue/green ABTS^•+^ chromophore.

ABTS was dissolved in water to a concentration of 7 mM. ABTS radical cation (ABTS^•+^) was produced by reacting ABTS stock solution with 2.45 mM potassium persulfate (final concentration) and allowing the mixture to stand in the dark at room temperature for 16 h before use. The ABTS^•+^ solution was diluted with a mixture of water: ethanol (1:1, v/v) until an absorbance of 0.7 ± 0.02 at 734 nm was reached. Twenty microliters of the extract were reacted with 1980 µL of ABTS^•+^ solution, and the absorbance was measured using a spectrophotometer (Agilent 8453, Agilent Technologies) at 734 nm after 6 min in the dark. To calculate the results, a standard curve was constructed using 25, 50, 100, 200, and 300 µmol/g concentrations of trolox (*y* = 0.338*x* + 2.7409, *R* ^2^ = 0.9959) and expressed as trolox (µmol trolox/g dry matter).

#### 2.3.3 Determination of total phenolic content

The TPC analysis was performed according to the method proposed by Singleton and Rossi (1965). Propolis extracts were diluted with methanol at an appropriate ratio, 0.4 mL was taken of 2 mL of Folin–Ciocalteu reagent diluted 1/10 with water, and 1.6 mL of 7.5% sodium carbonate solution was added and mixed. The tubes were incubated at room temperature in the dark for 1 h, and absorbance was measured at 760 nm using a spectrophotometer. TPC was calculated from the gallic acid calibration graph (*y* = 0.003*x* + 0.024, *R* ^2^ = 0.9982), and the results were expressed as gallic acid equivalents (mg GAE/100 g dry matter).

### 2.4 Analyzes on oil samples

#### 2.4.1 Determination of free fatty acidity (FFA)

FFA of the samples was performed according to AOCS Official Method Ca 5a‐40 (AOCS, 2004). Five grams of the oil sample were weighed, and 50 mL of an ethanol‐diethyl ether (1:1, v/v) mixture was added. Oil was completely dissolved by shaking. Add 5–6 drops of phenolphthalein (1%) solution was added and mixed. A 0.02 N ethanol sodium hydroxide solution was titrated until a pink color appeared. Free fatty acid content was calculated as a percentage of oleic acid using the formula shown in the following equation:

(2) $$\begin{array}{ccc} \%\mathrm{FFA}\;\left(\mathrm{in}\;\mathrm{oleic}\;\mathrm{acid}\right) & = & \frac{V\;\times \;T\;\times \;M_{a}\;\times \;100}{m\;\times \;1000}\\ & & =\frac{V\;\times \;T\;\times \;28.2}{m} \end{array}$$

where %FFA is the percent free fatty acidity, *V* is the spent ethanol sodium hydroxide solution (mL), *m* is the sample weight (g), *M*_a_ is the molecular weight of oleic acid (282 g), and *T* is the normality of the sodium hydroxide solution with ethanol.

#### 2.4.2 Determination of peroxide value (PV)

PV was used to determine the primary oxidation products of the first stage of oxidation in the oils. PV should not exceed 10 meq O_2_/kg, which is the upper limit specified in the Turkish Food Codex Communiqué on Oils with Plant Names (TGK, 2012). PV was determined according to the AOCS Official Method Cd 8‐53 (AOCS, 1989a). A volume of 25 mL acetic acid/chloroform (3/2. v/v) solution was added to 2 g of the oil sample and mixed thoroughly. Next, 1 mL of saturated potassium iodide (KI) solution was added, shaken for 1 min, and kept in the dark for 10 min. Then, 50 mL of pure water and 1 mL of starch solution were added and the sample was titrated with 0.01 N sodium thiosulfate. PV was calculated with the following equation:

(3) $$\mathrm{PV}=\frac{V\;\times T\;\times 1000}{m}$$

where PV is the peroxide value (meq O_2_/kg oil), *V* is the spent sodium thiosulfate solution (mL), *T* is the normality of the sodium thiosulfate solution, and *m* is the sample weight (g).

#### 2.4.3 Determination of conjugated‐diene (K_232_) and conjugated‐triene (K_270_)

Conjugation of fatty acids was determined according to AOCS Official Method Ch 5‐91 (AOCS, 1989b). The oil sample (0.25 g) was weighed into a 25 mL balloon jug, added to 25 mL of isooctane, and stirred well to dissolve the oil. The samples were read against the reference (pure iso‐octane) in a spectrophotometer (Agilent 8453, Agilent Technologies) at wavelength of 232–270 nm, respectively. The specific absorption values were calculated using the following equation:

(4) $$E_{1\;\mathrm{cm}}^{\%1}=K_{\lambda }=\frac{A_{\lambda }}{c\times \;l}$$

where *K_λ_* is the specific absorption wavelength, *A_λ_* is the absorbance at wavelength, *c* is the concentration of the solution (g/100 mL), and *l* is the length of the quartz cuvette (cm).

#### 2.4.4 Determination of p‐anisidine value (p‐AV)

The oil sample (0.5 g) was placed into a 25 mL volumetric flask and completed with hexane. The absorbance (*A*_1_) at 350 nm was read against the absorbance of the reference cell filled with hexane without a sample in a spectrophotometer (Agilent Technologies, Cary 60, UV–Vis). Five grams of oil (*m*) were placed in a test tube. One milliliter p‐AV was added to the test tube (0.25 g/100 mL glacial acetic acid). After 10 min, the absorbance (*A*_2_) of the sample was read at 350 nm against at reference cell prepared with hexane and p‐AV without the sample. The p‐AV value was calculated using the following equation (AOCS, 1998):

(5) $$p-\mathrm{AV}=\frac{25\;\times \;\left(1.2\;A_{2}A_{1}\right)}{m}$$

where *m* is the sample weight (g).

#### 2.4.5 Total oxidation value (TOTOX)

The TOTOX was determined according to the following equation (Stauffer, 1996):

(6) TOTOX=(2×PV)+p−AV

#### 2.4.6 Determination of fatty acid composition

Fatty acid composition analysis was performed using GC/MS (Shimadzu GC/MS QP 2010) by forming fatty acid methyl esters (FAMEs) (IUPAC, 1992). For this purpose, the methyl esters of each oil sample were prepared. An amount of 0.4 g of the oil sample was weighed, and 4 mL of iso‐octane was added. The mixture was thoroughly stirred, and 0.2 mL of potassium hydroxide (KOH) prepared in methanol was added, 6 min in the dark. Then 2–3 drops of methyl orange were added to the mixture, and 0.5 mL of 1 normal HCl solution was added. Finally, the mixture was kept in the dark for 30 min, and the clear liquid formed at the top was placed in vials. An Agilent DB‐23 (30 m long, 0.25 mm inner diameter, 0.25 µm film thickness) capillary column was used in the study. The operating conditions of the instrument were set as follows: column: DB‐23 (60 × 0.25 mm^2^, 0.25 µm), carrier gas: helium, total flow: 36.6 mL/min, column flow: 0.66 mL/min, linear speed: 21.2 cm/s, split rate: 50, initial temperature: 80°C, temperature program: 10°C/min, final temperature: 220°C, injection temperature: 250°C, detector temperature: 250°C, ion source temperature: 200°C, and total analysis time: 34 min. FAMEs were determined by comparing the retention time and equivalent chain length of FAMEs with standard FAMEs (47,885‐U, Supelco). The percentage area of FAMEs in the samples was used for quantification (AOAC, 1990).

### 2.5 Statistical analysis

SPSS (version 20.0, SPSS Inc.) was used to evaluate the results. One‐way analysis of variance (ANOVA) was used to analyze whether there was a statistically significant difference between the group averages. Significant differences between means were determined by Duncan's multiple range tests. *p* values less than 0.05 were considered statistically significant (*p* < 0.05).

## 3 RESULTS AND DISCUSSION

### 3.1 Antioxidant capacity of propolis

#### 3.1.1 DPPH radical scavenging activities

**FIGURE 2:**
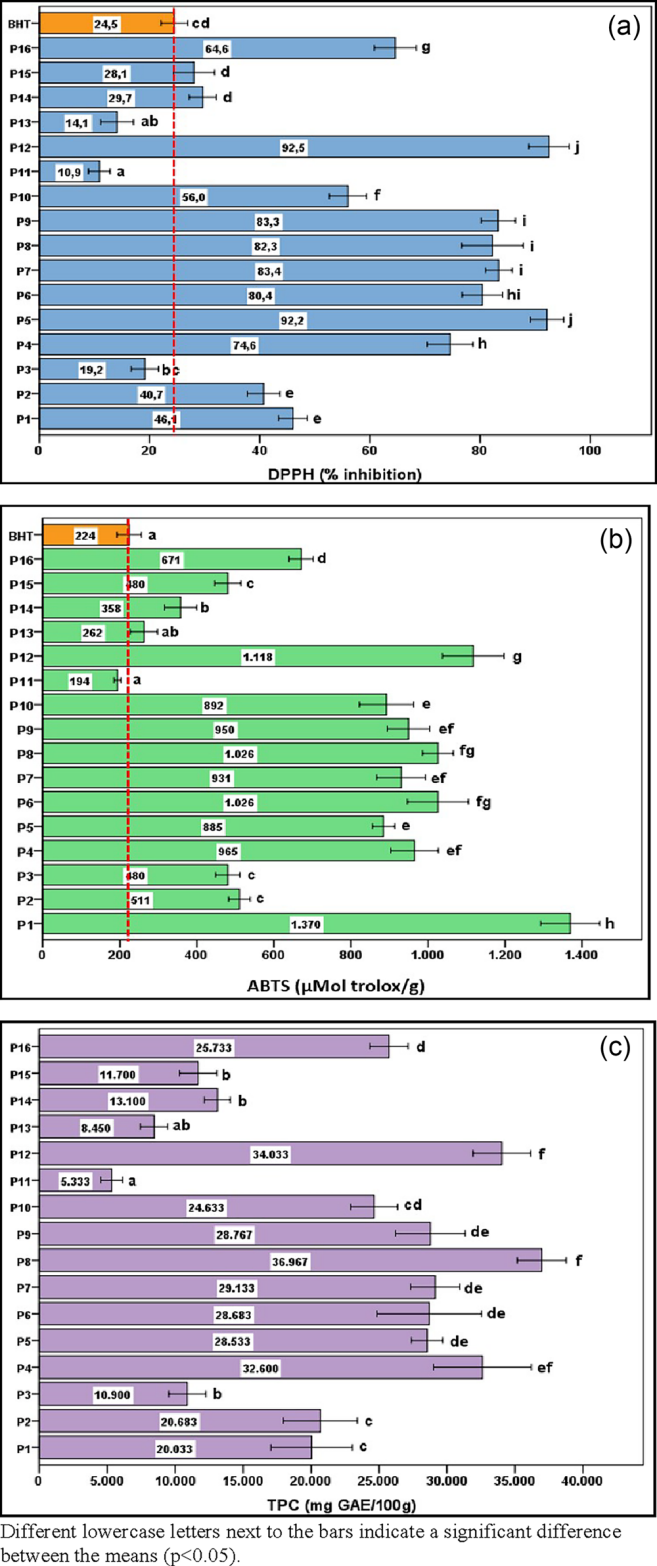
Comparison of antioxidant properties of propolis samples: (a) 1,1‐diphenyl‐2‐picrylhydrazyl (DPPH) inhibition (%), (b) 2,2′‐azino‐bis(3‐ethylbenzothiazoline‐6‐sulfonic acid (ABTS) values (µmol trolox/g), and (c) total phenolic content (TPC, mg GAE/100 g).

Numerous methods can be used to evaluate the radical‐scavenging capacities of natural and artificial compounds. Among of these, methods using stable DPPH and ABTS radicals are widely used because they are simple and require a relatively short time compared to other methods (Wang et al., 2004). However, according to Roginsky and Lissi (2005), it is recommended to avoid generalizing indirect methodologies for measuring potential antioxidant activity. In addition, as explained by Yehye et al. (2015), it should be noted that some antioxidants, such as BHT, may not interact sufficiently with the DPPH radical due to steric impediment failure and therefore may show low antioxidant activity values. In particular, the misinterpretation of the “effective concentration” (EC50) as a parameter related to the antioxidant properties of chemicals blurs the interpretation of the data drawn from it (Foti, 2015). Attention should also be paid to this aspect. The DPPH inhibition rates determined in propolis samples obtained from 16 different regions are shown in Figure 2a. The % inhibition value indicates how much of the DPPH radical is neutralized compared to the initial concentration. A high % inhibition value indicates strong antioxidant activity. These rates vary between 10.9% and 92.5% (*p* < 0.05). In statistics, “*p* < 0.05” indicates that the results obtained are statistically significant. This means that the probability of the observed results occurring by chance is less than 5%. Propolis with the P_12_ and P_5_ codes showed the highest antioxidant activity. This can probably be attributed to the diversity of trees in the region where the propolis was collected. These regions are the hottest places in Türkiye. This was followed by P_7_, P_9_, P_8_, and P_6_. The DPPH inhibition of these propolis samples ranged from 80.4% to 83.4%, whereas the DPPH inhibition of BHT was 24.5%. The inhibition rates of P_3_, P_11_, and P_13_ were lower than that of BHT. Sarıkahya et al. (2021) determined that the DPPH activities of 39 Turkish propolis extracts ranged between 55.98% and 86.17%. DPPH inhibition rates were reported as 25%–85% in Egyptian propolis (A. A. Mohdaly et al., 2015), 65%–94% in Korean propolis (Ahn et al., 2004), 7.82%–91.84% in Chilean propolis (Nina et al., 2015), 49.5%–65.7% in Argentine propolis (Chaillou & Nazareno, 2009), and 65% and 79% in Canadian propolis (Christov et al., 2006). A wide DPPH variation (10.9%–92.5%) obtained in the study is in agreement with previous studies. As the propolis samples were collected from different locations in Türkiye, the chemical compounds varied accordingly. These components may vary according to the botanical and geographical origin of propolis. Considering that propolis obtained from different phytogeographic regions of Türkiye exhibits different chemical profiles, the DPPH variations (*p* > 0.05) detected between the samples were expected.

#### 3.1.2 ABTS^•+^ scavenging ability

ABTS values in propolis samples varied between 48.5 and 342.8 mg trolox/g (193.7–1369.6 µmol trolox/g) (*p* < 0.05) (Figure 2b). In BHT, this value was 224 µmol trolox/g. The value of P11 was lower than that of BHT. The highest ABTS values were found in samples P_1_ and P_12_, and the lowest value was found in sample P_11_. No significant differences were found between the ABTS values at P_2_, P_3_, and P_15_ and P_4_, P_7_, and P_9_. This is probably due to the fact that the locations where these samples were collected have similar environmental conditions and similar botanical origins of the flora. ABTS values were determined as 497.8, 503.7, 448.9, and 409.6 mg trolox/g in propolis obtained from important honey production areas of Türkiye such as Bingöl, Rize, Tekirdağ, and Van (Erdogan et al., 2011). Degirmencioglu et al. (2019) determined ABTS values in the range of 24.94–309.81 mg trolox/g in 19 different crude propolis samples collected from five different regions of Türkiye (Aegean, Black Sea, Central Anatolia, Marmara, Mediterranean Regions). In the study conducted by Mohtar et al. (2020), antiradical activities (ABTS) in Venezuelan, Argentine, and Brazilian propolis were determined as 13.01–102.3, 107.3–108.0, and 105.3 mg trolox/g, respectively. In another study, ABTS values in Brazilian propolis were determined in the range of 99.78–384.60 mg trolox/g (Cristina et al., 2016). Our results are consistent with most of these findings. The reason why the data in the literature partially differ from our results may be attributed to differences in the flora, climate, or collection methods of propolis.

#### 3.1.3 Total phenolic content (TPC)

TPC, which is considered to be one of the factors contributing to antioxidant activity, is shown in Figure 2c. TPCs were found in the range of 5333–36,967 mg GAE/100 g (*p* < 0.05). The lowest TPC was detected in P_11_, whereas the highest TPC was detected in P_8_. Although there was no statistically significant difference between the TPCs of samples P_5_, P_6_, P_7_, and P_9_, a significant difference was found between the other samples (*p* < 0.05). This may be attributed to the fact that these regions where the samples were collected are generally located in the west of Turkey and have similar geographical characteristics. TPCs were determined in the range of 1124–17,298 mg GAE/100 g in 19 different crude propolis samples collected from five different regions of Türkiye (Aegean, Black Sea, Central Anatolia, Marmara, and Mediterranean Regions) (Degirmencioglu et al., 2019). In previous studies, TPCs was reported to range from 21,188 to 22,762 mg GAE/100 g in Spanish propolis (Oses et al., 2016), 15,005–19,714 mg GAE/100 g in Polish propolis (Socha et al., 2015), and 1910–10,700, 17,600–18,200 and 15,800 mg GAE/100 g in Venezuelan, Argentinian, and Brazilian propolis, respectively (Mohtar et al., 2020). Because the TPC method used in our study is consistent with the cited studies, the differences in the results are not methodological. The differences in the results are probably due to the flora of the location where the propolis was collected, climatic factors, and collection methods.

### 3.2 Correlation between DPPH, ABTS, and TPC

The correlation between DPPH, ABTS, and TPC contents determined in the propolis samples is given in Table S1. As depicted in the table, it can be concluded that a strong positive relationship exists between these three variables. The high positive correlation between TPC and DPPH (*r* = 0.936, *p* < 0.01) and between TPC and ABTS (*r* = 0.786, *p* < 0.01) demonstrates the significant role of phenolic content in antioxidant activity. Similarly, the correlation between DPPH and ABTS (*r* = 0.796, *p* < 0.01) indicates consistency across different antioxidant assays. Degirmencioglu et al. (2019) observed very strong positive significant correlations between TPC values and ABTS (*r* = 0.949) and DPPH (*r* = 0.953) in Anatolian propolis. Altuntaş et al. (2023) observed a strong positive correlation between DPPH and TPC (*r* = 0.80) in propolis collected from different regions of Türkiye. Kumazawa et al. (2010) reported the correlation coefficient between DPPH and TPC of propolis collected from different regions of Argentina as 0.837. Papachristoforou et al. (2019) found a strong positive correlation between antioxidant activity and TPC in propolis from Samothraki Island, Greece (0.912). These findings are largely consistent with our results.

### 3.3 Peroxide value (PV)

**FIGURE 3:**
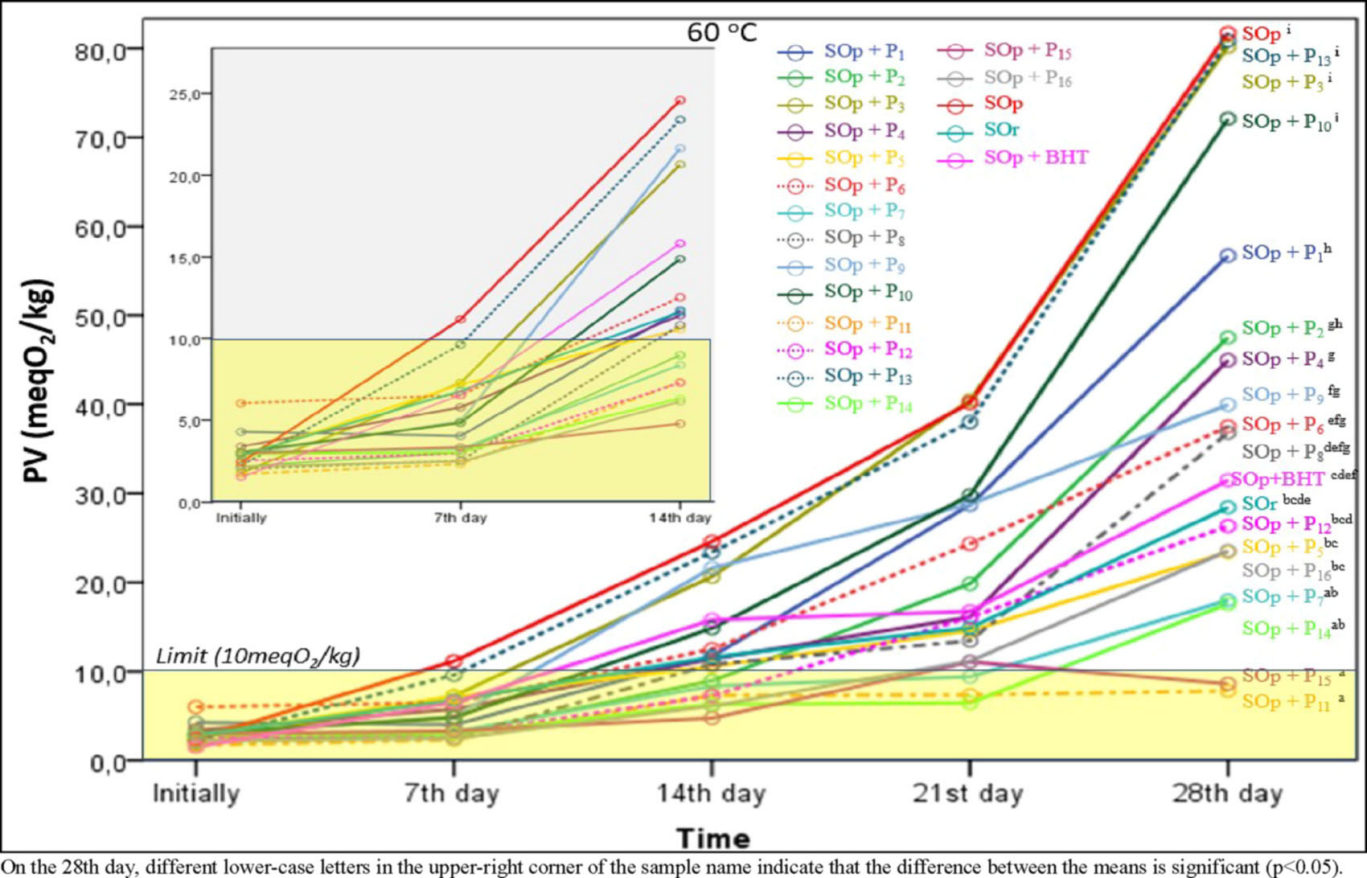
Change in peroxide value (PV) of samples during storage at 60°C for 28 days.

PV is the most widely used method for determining the primary oxidation products of the first stage of oxidation in oils. In our previous studies (Basturk et al., 2007; Javidipour et al., 2015), we observed that the oxidation evolution at 60°C was as expected; therefore, the temperature was set to 60°C for accelerated oxidation conditions. The PVs of sunflower oils with added propolis extract under accelerated oxidation conditions (60°C) on the initial, 7th, 14th, 21st, and 28th day are given in Figure 3. The PVs determined in the oil samples ranged from 1.50 to 81.75 meq O_2_/kg (*p* < 0.05). Before undergoing accelerated oxidation (initially), PVs were in the range of 1.50–6.00 meq O_2_/kg. These values were within the regulatory limits (10 meq O_2_/kg) (*p* < 0.05). In all the samples, the PV generally increased with storage time. On the 28th day, the PV in the BHT‐containing oil sample increased approximately 21‐fold compared with the initial value (from 1.50 to 31.50 meq O_2_/kg). ANOVA was performed for the values obtained on the 28th day of storage. The highest PVs were in SOp + P_3_, SOp + P_10_, SOp + P_13_, and SOp, and there was no significant difference between them. Especially in the SOp + P_11_, SOp + P_15_, SOp + P_14_, and SOp + P_7_ samples, propolis extracts significantly reduced the PV increase. There was no significant difference between SOp + P_11_ and SOp + P_15_ and between SOp + P_14_ and SOp + P_7_. The difference between the PVs of the other samples on Day 28 was significant (*p* < 0.05). The reason for these differences in PVs may be the different phenolic compounds present in the propolis samples. On the seventh day of storage, the PVs did not exceed the limit, except for the SOp sample. This was expected in the SOp sample, which did not contain antioxidants. In most samples, the PVs exceeded the set limit (10 meq O_2_/kg) after Day 14. In particular, P_11_, P_15_, P_14_, and P_7_ propolis extracts significantly slowed the PV increase after the 14th day. A possible reason for this may be that they contain high concentrations of effective phenolic compounds. There was no statistically significant difference in the PVs of sunflower oil samples containing P_2_, P_12_, P_14_, and P_15_ until the 14th day of storage at 60°C. On the 21st day of storage, PVs were above the legal limit in all samples, except SOp + P_14_, SOp + P_11_, and SOp + P_7_. On the 28th day of oxidation, the highest PVs occurred in SOp (81.75 meq O_2_/kg), SOp + P_13_ (80.90 meq O_2_/kg), and SOp + P_3_ (80.20 meq O_2_/kg). On the 28th day, the PVs were ordered according to efficacy as follows: P_11_ > P_15_ > P_14_ > P_7_ > P_5_ > P_16_ > P_12_ > BHT. Ceylan and Baştürk (2022) reported the antioxidant effects of BHT and natural extracts against peroxide formation as a result of the frying process in palm oil as BHT > propolis > quinoa > uşkun. In a study in which propolis extract was used as a natural antioxidant in fish oils, propolis extract and BHT (100 mg/kg) at concentrations of 100, 500, and 1000 mg/kg were added to fish oil samples, and oxidation levels were determined. During storage, the PV, K_232_, K_270_, TBARS, and p‐AV values were lower than those of the control and BHT‐containing groups. The results showed that 1000 mg/kg was the most effective dose (Uçak, 2018). Osman and Taha (2008) subjected sunflower oil to rapid oxidation at 63°C for 4 days after adding propolis extracts, BHT, and TBHQ. They reported that 200 and 300 ppm propolis extracts were better than BHT at 300 ppm, but lower than TBHQ at 200 ppm. Özcan (2000) reported that propolis extracts were more effective than BHT and BHA at 0.01% concentration in preventing peroxide formation in olive oils stored at 60°C to which propolis extracts were added at 0.04%–0.08% concentrations.

### 3.4 p‐Anisidine value (p‐AV)

**FIGURE 4:**
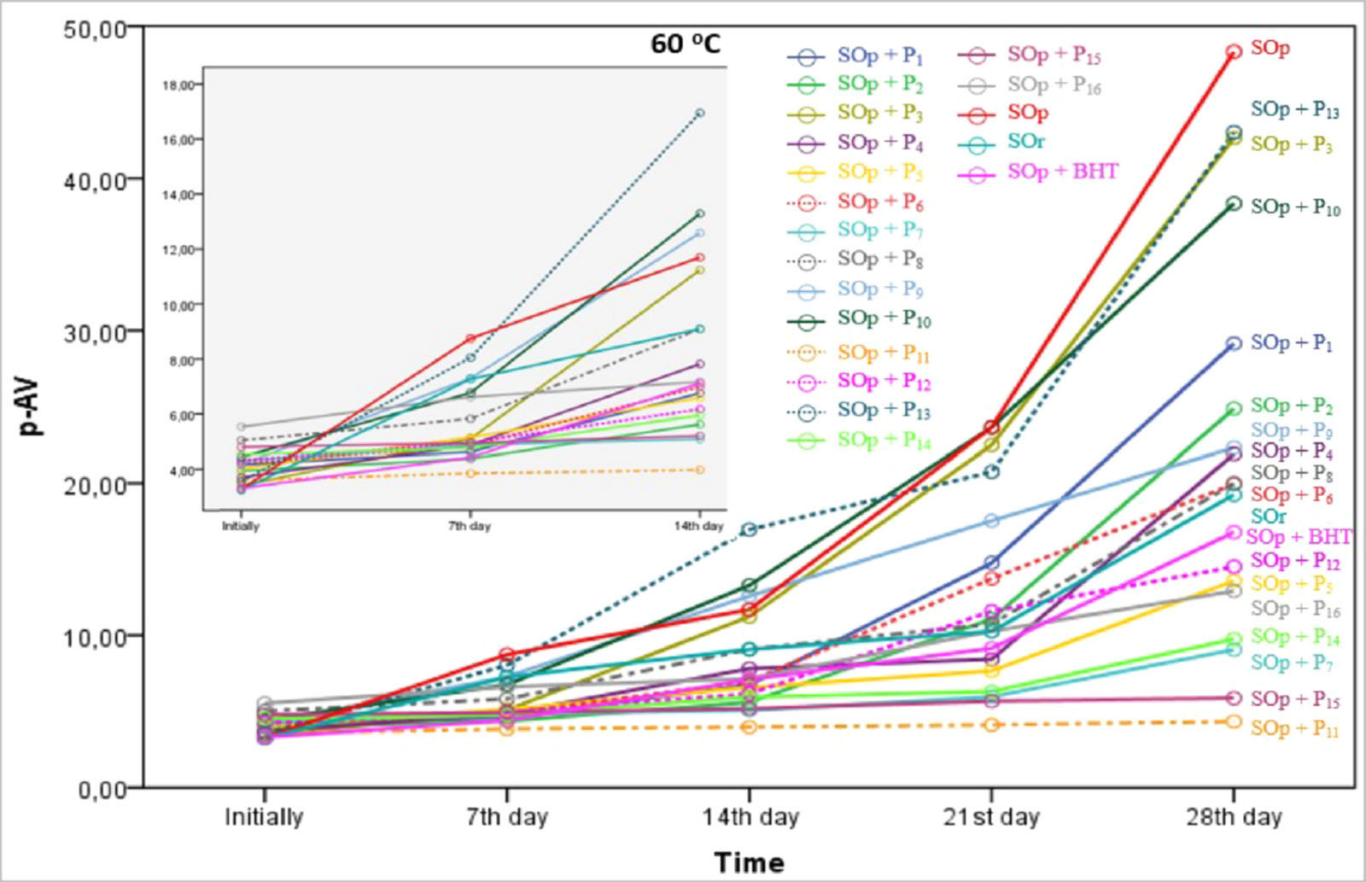
Change in p‐anisidine (p‐AV) of samples during storage at 60°C for 28 days.

p‐AV represents the content of secondary oxidation products such as α‐ and β‐alkenals, and all compounds that can react with the p‐AV reagent. The p‐AV changes in the samples during storage are shown in Figure 4. The p‐AVs, which ranged between 3.27 and 5.53 at the beginning of storage, increased on the 28th day of oxidation and reached values ranging between 4.34 and 48.32 (*p* < 0.05). Until the 14th day of oxidation, p‐AVs did not show a statistically significant increase in the samples with P_1_, P_2_, P_6_, P_7_, P_12_, and P_16_ additions. This can probably be explained by the fact that these propolis extracts exhibit antioxidative properties during advanced stages of oxidation (secondary oxidation). Compared to baseline values, on Day 28 of oxidation, p‐AVs increased approximately 14‐fold in SOp, 12‐fold in SOp + P_3_, and SOp + P_13_, 9‐fold in SOp + P_10_, 7‐fold in SOp + P_1_, 6‐fold in SOp + P_2_, SOp + P_4_, and SOr, and 5‐fold in SOp + P_6_, SOp + P_9_, and SOp + BHT. Propolis extracts P_11_, P_15_, P_7_, P_14_, P_16_, P_16_, P_5_, and P_12_ added to sunflower oil at a concentration of 1000 ppm were more effective than BHT added at a concentration of 200 ppm in inhibiting or slowing down p‐AV formation (Figure 4). It is understood that propolis extracts P_13_, P_3_, and P_10_ did not have significant effects in preventing the formation of p‐AV, the secondary product of oxidation. It can be said that propolis codes P_11_, P_15_, P_7_, and P_14_ significantly inhibited or slowed down the formation of p‐AV. As shown in Figure 4, the p‐AV and PV formation in the samples showed a similar trend. This can be interpreted as a significant antioxidative effect of this propolis under the present conditions. Mohdaly et al. (2010) used sunflower oil samples to accelerated oxidation at 70°C for 72 h with different extracts (potato peels and sugar beet pulp) and synthetic antioxidants (TBHQ, BHT, and BHA). p‐AV was 0.98 at the beginning and 14.2, 9.10, 9.40, 9.42, 9.36, and 8.84 after 72 h in the control, potato peel extract with SO, sugar beet pulp extract with SO, BHA, BHT, and TBHQ with SO, respectively. These results obtained at the end of the incubation period corresponding to three days were higher than our findings. This may be due to the storage temperature being 10°C higher than ours and the antioxidant substances being different. These values were close to the values we obtained on the 7th day. In another study, sunflower oil samples supplemented with α‐ and δ‐tocopherol, citric acid, ascorbic acid, and ascorbyl palmitate were stored at 30°C for 35 days, 68°C for 23 days, and 130°C for 1 day, and p‐AVs were found in the ranges of 2.72–2.89, 10.4–11.7, and 30.0–60.0, respectively (Carelli et al., 2005). The values obtained after 23 days of storage at 68°C, which is close to our storage temperature (10.4–11.7), were lower than some of our values obtained on the 21st day (SOp, SOp + P_3_, SOp + P_13_, SOp + P_10_, and SOp + P_9_). This shows that the different antioxidant substances used in this study were more effective. Mei et al. (2014) determined the maximum p‐AVs in the range of 5.04–6.98 in sunflower oils with different antioxidants subjected to accelerated oxidation at 60°C for 24 h. These values are quite low according to our results. This is normal because they are p‐AVs formed after 1 day of storage. Similar results have been obtained in previous studies (Chen et al., 2014; Costa et al., 2013; Mitra et al., 2012; Yim et al., 2013; Zhang et al., 2010). The main reasons for the differences between the abovementioned studies and our study results may be attributed to the different antioxidant substances and their concentrations used, and the different storage temperatures and durations of the oils.

### 3.5 TOTOX

**FIGURE 5:**
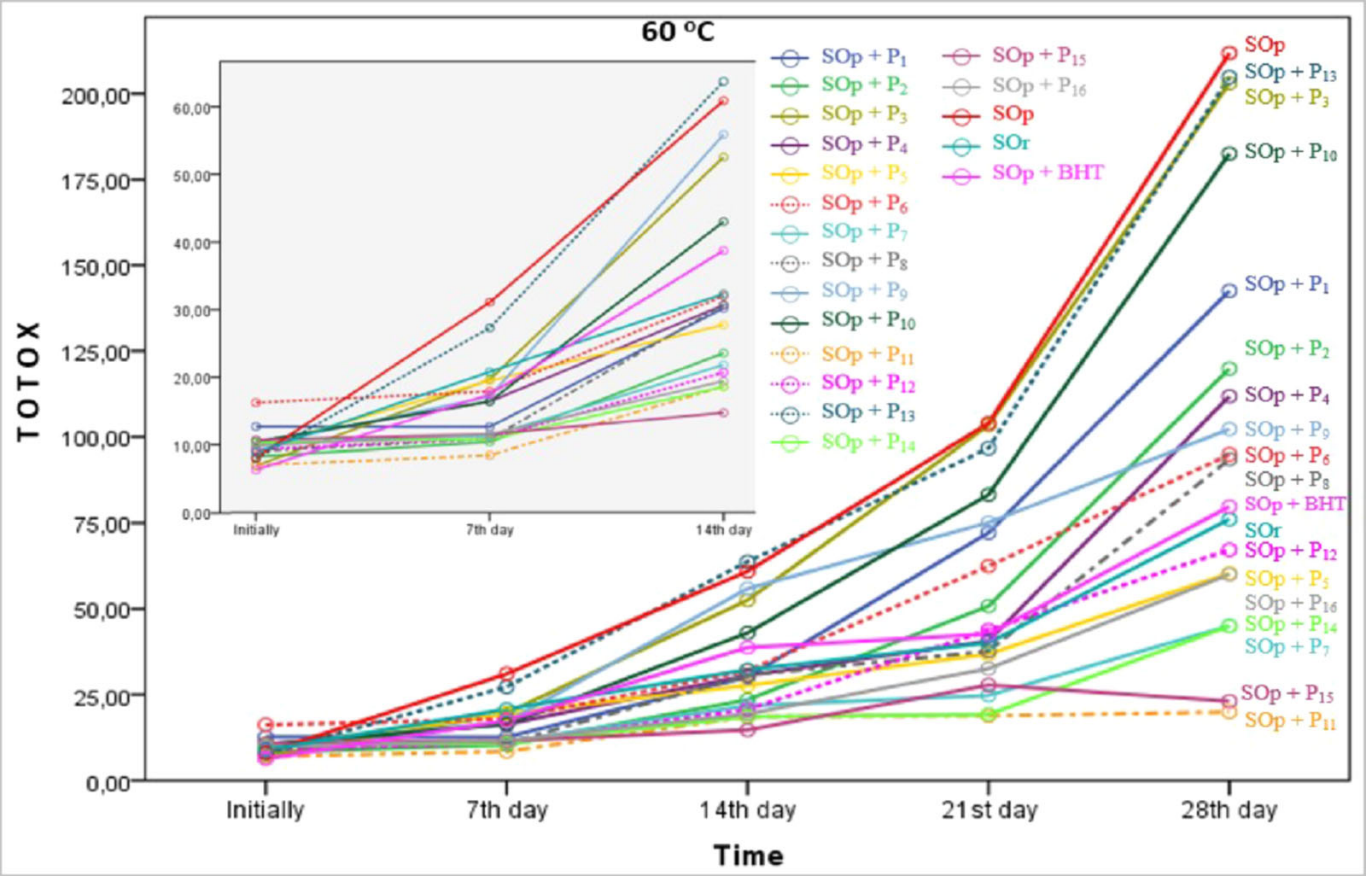
Change in total oxidation (TOTOX) of samples during storage at 60°C for 28 days.

TOTOX analysis is an important method for assessing the oxidative stability and freshness of oils. The TOTOX represents the sum of the primary (PV) and secondary (p‐AV) oxidation products. This value assesses the oxidative status and potential degradation of oils. High TOTOX values indicate that the oil was oxidized and possibly degraded. The TOTOX in edible oils was calculated from PV and p‐AV (2PV + p‐AV). This value suggests that the peroxides were formed as a result of oxidation and the aldehydes and ketones were released in the next step (Sun et al., 2011). The changes in TOTOX values of the oil samples during the storage period are shown in Figure 5. These values varied between 6.30 and 16.20 at the initially and 19.94 and 211.82 on the 28th day (*p* < 0.05). As expected, the TOTOX value exhibited the highest increase in the SOp. In the SOp sample, the TOTOX increased by approximately 26 times compared with the initial value and reached 211.82. This is followed by the SOp + P_13_ and SOp + P_3_ samples. It can be concluded that the P_13_ and P_3_ coded propolis did not show antioxidant effects under these conditions. After 21 days of storage, the content of most of the samples increased dramatically. When TOTOX was considered, propolis P_11_, P1_5_, P_14_, P_7_, P_16_, P_5_, and P_12_ added to sunflower oil at a concentration of 1000 ppm were more effective than BHT added at a concentration of 200 ppm. These propolis extracts probably contain compounds that inhibit or slow down the formation of both primary and secondary oxidation products. In particular, P_15_ and P_11_ (at 1000 ppm) were 4–6 times more effective than BHT (at 200 ppm) in inhibiting TOTOX formation in sunflower oil. Okhli et al. (2020) investigated the oxidation stability of aqueous, ethanolic, and methanolic extracts of citron peel (*Citrus medica* L.) by adding 800, 200 ppm BHT, and citron peel essential oil at 800 ppm to sunflower oil samples stored at 65°C for 24 h. TOTOX was determined between 8.40 and 18.20 at the beginning and 50.21 and 86.41 at the end of 5 days. Our values on Day 7 were lower than these findings. This may indicate that propolis is a more effective antioxidant in sunflower oil. Zaborowska et al. (2012) stored sunflower oil to which they added 1% ethanol extract of thyme for 29 days at 38°C. TOTOX, which was 1.48 at the beginning, increased to 63.80 in pure sunflower oil and 25.82 in the antioxidized sample. Mei et al. (2014) subjected sunflower oil samples to accelerated oxidation at 60°C for 24 days with the addition of different antioxidant substances and determined TOTOX in the range of 62.08–304.35. Our results were consistent with these findings. Additionally, our results are consistent with the findings of previous studies by Chinprahast et al. (2016) and Rege et al. (2014).

### 3.6 Conjugated dienes (K_232_) and trienes (K_270_)

K_232_ and K_270_, expressed as specific extinction coefficients, indicate the oxidation state of the oil, providing information on the quality, state of preservation, or changes caused by technological processes. The specific extinction coefficient measured at 232 nm (K_232_) is related to hydroperoxides, which are the primary products of fat oxidation, and conjugated dienes, which are formed during the intermediate stage of fat oxidation. However, at 270 nm (K_270_), this coefficient value has been reported to be related to carbonyl compounds (secondary stages of fat oxidation) and conjugated trienes (Guzmán et al., 2011; Muik et al., 2005). As a result of the oxidation reactions, geometric isomerization occurs in the parts of polyunsaturated fatty acids containing methyl groups, and the location of the double bonds changes with this event. Thus, the structure of the fatty acid, which is the change in the location of the double bond, changes to a conjugated structure. The conjugated structure formed in fatty acids is important because it is more prone to oxidative reactions than the isolated structure and leads to drying reactions in oil in the presence of oxygen. The increase in conjugated diene formation during oxidative reactions is considered an important parameter as it provides information about the degradation status of oils. K_232_ values determined for sunflower oil samples subjected to accelerated oxidation (60°C, 28 days) are presented in Table 1. K_232_ formation increased with increasing storage time. The majority of the added propolis maintained an increase in K_232_ at a lower level than the control samples SOp and SOr. The K_232_ values initially varied between 1.973 and 3.620 (*p* < 0.05). These findings coincide with the results obtained for seven different brands of sunflower oil (2.74–4.97) in a previous study (Gündüz & Baştürk, 2023). The highest K_232_ values were recorded at the end of the storage period on Day 28 (*p* < 0.05). At the end of this period, the highest values were determined in the SOr, SOp + P_3_, SOp + P_10_, SOp + P_13_, and SOp samples. In this case, this result can be expected for SOr and SOp, as propolis extract was not added. This can be attributed to the fact that extracts P3, P10, and P13 are inert and contain prooxidant substances. Günal and Turan (2018) determined K_232_ values in sunflower oils to which they added different antioxidant substances subjected to accelerated oxidation at 60°C in the ranges of 3.25–403 and 17.00–98.25 at the beginning and 21st day, respectively. Our results were lower than those of these previous findings. This is probably due to the differences in the antioxidants used and the initial oil quality. In other words, it can be attributed that propolis extracts have more effective antioxidant properties. Budryn et al. (2011) measured the K_232_ values of sunflower oils containing which different coffee bean extracts were added after 12 weeks of storage at 20°C. Although K_232_ increased from 3.0 to 23.1 in the control sample, it increased from 6.0 to 14.3 in the extract‐supplemented samples. In SOp + P_1_, SOp + P_2_, SOp + P_7_, SOp + P_8_, SOp + P_9_, SOp + P_10_, and SOp + P_14_ samples, K_232_ values did not show a statistically significant increase until the 14th day, whereas they showed a significant increase in the following storage periods (21st and 28th days) (*p* < 0.05).

**TABLE 1:** K_232_ values of sunflower oil samples with different propolis extracts and butylated hydroxytoluene (BHT), measured at various storage periods at 60°C.

Sample	Initially	7th day	14th day	21st day	28th day
SOp + P_1_	2.295±0.085^cdefA^	2.745±0.095^gA^	3.258±0.105^gA^	5.263±0.704^hıjB^	6.757±0.729^efC^
SOp + P_2_	2.200±0.092^bcdA^	2.460±0.037^defA^	2.713±0.122^cdA^	4.107±0.5^efgB^	6.919±0.846^efgC^
SOp + P_3_	2.253±0.039^cdeA^	2.648±0.091^fgAB^	4.073±0.07^ıB^	6.050±0.806^jC^	9.657±1.012^hD^
SOp + P_4_	2.335±0.177^defA^	1.638±0.022^aA^	3.330±0.027^ghB^	3.750±0.389^defgB^	6.363±0.723^deC^
SOp + P_5_	2.075±0.072^abcA^	2.223±0.142^cdAB^	2.738±0.095^cdBC^	3.075±0.31^bcdeC^	4.969±0.396^cdD^
SOp + P_6_	2.395±0.03^defAB^	2.283±0.054^cdA^	3.015±0.14^efBC^	3.344±0.485^bcdefC^	4.744±0.315^bcD^
SOp + P_7_	2.075±0.054^abcA^	2.318±0.134^cdA^	2.380±0.085^aA^	2.600±0.171^abcdA^	3.413±0.47^abcB^
SOp + P_8_	2.395±0.024^defA^	2.305±0.136^cdA^	2.473±0.043^abA^	3.257±0.233^bcdefB^	4.519±0.571^bcC^
SOp + P_9_	2.248±0.121^cdeA^	2.613±0.146^fgA^	3.178±0.043^fgA^	4.382±0.359^fghB^	7.063±0.886^efgC^
SOp + P_10_	3.225±0.151^gA^	2.428±0.097^defA^	3.498±0.1^hA^	4.750±0.334^ghıB^	9.613±0.92^hC^
SOp + P_11_	2.323±0.09^defAB^	1.995±0.006^bA^	2.420±0.127^aAB^	2.544±0.214^abcB^	2.782±0.286^aB^
SOp + P_12_	2.515±0.122^fA^	2.310±0.122^cdA^	2.723±0.127^cdAB^	3.169±0.301^bcdeB^	3.875±0.313^abcC^
SOp + P_13_	2.515±0.075^fA^	2.723±0.114^gA^	4.485±0.038^jB^	5.600±0.348^ıjC^	8.438±0.668^ghD^
SOp + P_14_	1.983±0.039^abA^	2.123±0.107^bcA^	2.455±0.061^aA^	2.207±0.211^abA^	2.413±0.333^aA^
SOp + P_15_	2.258±0.152^cdeAB^	2.373±0.047^deBC^	2.683±0.042^bcdC^	1.919±0.132^aA^	3.157±0.257^abD^
SOp + P_16_	2.393±0.07^defA^	2.555±0.069^efgAB^	2.558±0.093^abcAB^	3.094±0.294^bcdeB^	3.788±0.337^abcC^
SOp	2.478±0.062^efA^	3.285±0.03^hAB^	4.270±0.071^ıB^	7.225±0.764^kC^	8.182±0.651^fghC^
SOr	3.620±0.127^hA^	4.540±0.098^ıA^	7.325±0.185^kB^	9.757±1.217^lC^	14.057±1.521^ıD^
SOp + BHT	1.973±0.069^aA^	2.373±0.158^deAB^	2.850±0.091^deB^	3.657±0.301^cdefgC^	4.607±0.398^bcD^

*Note*: Values represent the specific extinction coefficients at 232 nm (K_232_), indicating the presence of hydroperoxides and conjugated dienes. Different lowercase letters given as exponents in the same column indicate a significant difference between the means (*p* < 0.05). Different capital letters given as exponents in the same row indicate a significant difference between the averages depending on the duration (*p* < 0.05).

The K_270_ values of the samples are listed in Table 2. These values varied between 0.978 and 1.593 at the beginning and between 0.844 and 1.663 on the 28th day (*p* < 0.05). K_270_ values fluctuated depending on the storage period. The K_270_ values in the samples other than SOr varied between the 0.844 and 1.420 band (*p* < 0.05). Interestingly, most samples showed lower K_270_ levels on Day 28 than at baseline. This is likely due to the conversion of polyunsaturated fatty acids into monounsaturated or saturated fatty acids as a result of the increase in temperature and time. In a study using different antioxidants in sunflower oils, K_270_ values were determined to be in the ranges of 2.86–3.36 and 2.49–7.48 at baseline and Day 21, respectively (Günal & Turan, 2018). Our results were lower than these findings. This shows that the propolis extracts we used can be more effective.

**TABLE 2:** K_270_ values of sunflower oil samples with different propolis extracts and butylated hydroxytoluene (BHT), measured at various storage periods at 60°C.

Sample	Initially	7th day	14th day	21st day	28th day
SOp + P_1_	1.178±0.006^bcdefgB^	1.248±0.028^deBC^	1.185±0.023^abcdeB^	1.363±0.03^hC^	0.975±0.091^bcA^
SOp + P_2_	1.133±0.013^bcdefAB^	1.215±0.03^cdeBC^	1.078±0.021^abA^	1.238±0.066^defghC^	1.094±0.042^cdefA^
SOp + P_3_	1.230±0.044^defgB^	1.370±0.071^fgB^	1.345±0.062^fB^	1.588±0.081^ıC^	1.007±0.07^bcdA^
SOp + P_4_	1.220±0.115^cdefgB^	0.840±0.173^aA^	1.278±0.016^defB^	1.257±0.021^efghB^	1.157±0.083^efgB^
SOp + P_5_	1.080±0.059^abcdA^	1.158±0.025^cdeAB^	1.190±0.034^abcdeAB^	1.119±0.099^bcdeA^	1.307±0.081^hB^
SOp + P_6_	1.245±0.055^efgB^	1.188±0.012^cdeB^	1.115±0.144^abcAB^	1.057±0.066^abcAB^	0.982±0.015^bcA^
SOp + P_7_	1.045±0.062^abA^	1.238±0.067^deB^	1.100±0.027^abcAB^	1.094±0.059^abcdAB^	1.157±0.08^efgAB^
SOp + P_8_	1.220±0.02^cdefgB^	1.215±0.042^cdeB^	1.098±0.015^abcA^	1.275±0.052^efghB^	1.282±0.008^ghB^
SOp + P_9_	1.128±0.04^abcdefAB^	1.243±0.076^deB^	1.148±0.019^abcdAB^	1.169±0.038^cdefAB^	1.057±0.026^cdeA^
SOp + P_10_	1.300±0.146^ghA^	1.128±0.025^bcdA^	1.225±0.126^cdefA^	1.075±0.038^abcA^	1.138±0.042^defA^
SOp + P_11_	1.098±0.023^abcdeB^	1.015±0.033^bA^	1.073±0.021^aAB^	1.194±0.027^cdefgC^	1.213±0.043^fghC^
SOp + P_12_	1.420±0.068^hB^	1.193±0.019^cdeA^	1.180±0.023^abcdeA^	1.344±0.076^ghB^	1.119±0.024^defA^
SOp + P_13_	1.073±0.035^abcBC^	1.098±0.026^bcBC^	1.185±0.03^abcdeC^	1.000±0.139^abAB^	0.844±0.025^aA^
SOp + P_14_	1.108±0.115^abcdefA^	1.153±0.033^cdeA^	1.135±0.016^abcA^	1.125±0.083^bcdeA^	0.982±0.026^bcA^
SOp + P_15_	1.125±0.059^abcdefB^	1.275±0.052^efC^	1.300±0.025^efC^	0.944±0.069^aA^	1.332±0.013^hC^
SOp + P_16_	1.260±0.037^fgAB^	1.410±0.018^gC^	1.225±0.011^cdefA^	1.319±0.025^fghB^	1.213±0.03^fghA^
SOp	1.305±0.061^ghBC^	1.373±0.013^fgC^	1.213±0.03^bcdeB^	1.313±0.033^fghC^	0.900±0.033^abA^
SOr	1.593±0.013^ıA^	1.575±0.03^hA^	2.100±0.098^gC^	1.800±0.045^jB^	1.663±0.132^ıAB^
SOp + BHT	0.978±0.016^aA^	1.213±0.023^cdeB^	1.203±0.047^abcdeB^	1.200±0.069^cdefgB^	1.138±0.012^defB^

*Note*: Values represent the specific extinction coefficients at 270 nm (K_270_), indicating the presence of carbonyl compounds and conjugated trienes. Different lowercase letters given as exponents in the same column indicate a significant difference between the means (*p* < 0.05). Different capital letters given as exponents in the same row indicate a significant difference between the averages depending on the duration (*p* < 0.05).

Upadhyay and Mishra (2015) determined K_270_ values in the range of 0.72–1.21 in sunflower oil samples to which different antioxidant substances were added. These findings are consistent with our results.

### 3.7 Free fatty acid (FFA)

FFAs are among the compounds that are naturally present in low amounts in vegetable oils. Their amount, which is a product of the hydrolytic degradation of triglycerides, is considered an important quality index for oils. The FFAs found in samples stored for different storage periods at 60°C are shown in Table S2. The FFAs exhibited an irregular variation in the range of 0.056%–0.245%. FFAs showed fluctuating changes depending on the storage time in all samples, except SOp + P_5_, SOp + P_10_, and SOp + P_16_ (*p* < 0.05). The difference between the mean FFA values of the samples for all four storage periods was significant (*p* < 0.05). Initially, the FFA content of the samples was in the range of 0.080%–0.209%. The samples with the lowest FFA content during storage were SOp + BHT and SOp. There is no possibility of water in these samples. This value was higher in samples containing propolis extract. This suggests that there may be a small amount of water in the extracts, which may cause hybridization. Gündüz and Baştürk (2023) determined FFAs in the range of 0.13%–0.22% (in oleic acid) in seven different brands of sunflower oil sold in the Türkiye market. These data are consistent with our findings. According to Turkish Food Codex Communiqué on Oils Called by Plant Name (TGK, 2012, FFA (in terms of oleic acid, %) in edible sunflower oils should reach a maximum of 0.3. Accordingly, this limit value was not exceeded in any sample during 28 days of storage at 60°C. Chen et al. (2014) determined FFAs in the range of 0.20–0.46 mg/kg (0.10%–0.23% in oleic acid) in sunflower oils containing different antioxidant substances stored at 60°C for 21 days. In a similar study, Mei et al. (2014) determined FFAs in the range of 0.27–0.57 mg/kg (0.135%–0.285% in oleic acid) in sunflower oil samples subjected to accelerated oxidation at 60°C for 24 days with different antioxidants added. Our results are consistent with these findings. In general, it can be said that BHT, P_2_, P_3_, P_6_, P_8_, and P_13_ are effective on FFA. It should also be noted that some of the other propolis extracts increased the FFA levels.

### 3.8 Fatty acid composition

Fatty acid composition is very important for the oxidative stability, taste and odor, nutritional value, and shelf life of oils. The degree of unsaturation of fatty acids affects the stability of the oil against oxidation. Unsaturated fatty acids are more prone to oxidation, which can cause fat to deteriorate faster. Two important factors that determine the susceptibility of fats to oxidation are the fatty acid composition and minor anti‐ and prooxidant components. The types of fatty acids present in oils, particularly the number of double bonds, determine the type and extent of chemical reactions that occur during storage. Polyunsaturated fatty acids, such as C18:2 (linoleic acid) and C18:3 (linolenic acid), are more susceptible to autoxidation than monounsaturated fatty acids (C18:1, oleic acid). Normal sunflower oil is characterized by a high concentration of linoleic acid, followed by oleic acid. Saturated fatty acids (mainly palmitic and stearic acids) constituted no more than 15% of the total fatty acid content. The abundance of linoleic acid in sunflower oils, ranging from 50% to 60%, makes these oils more sensitive than some vegetable oils in terms of their oxidative stability. The sunflower oil samples that were exposed to accelerated oxidation at 60°C on the initial, 14th, and 28th days had their fatty acid compositions displayed in Table 3. The fatty acid composition of sunflower oil samples analyzed at the beginning of storage was determined to be myristic acid (0.30%–0.88%), palmitic acid (5.85%–6.27%), stearic acid (3.75%–4.08%), oleic acid (29.43%–30.96%), linoleic acid (57.89%–59.66%), and arachidic acid (0.13%–0.19%). Gündüz (2020) determined the fatty acid compositions as myristic acid (0.09%–0.13%), palmitic acid (6.63%–7.53%), stearic acid (3.91%–4.52%), oleic acid (34.61%–44.85%), linoleic acid (42.59%–51.35%), and arachidic acid (0.13%–0.19%) in seven different sunflower oil samples sold in Türkiye. Although our findings were in parallel with those except for linoleic acid, the linoleic acid ratio was found to be higher in the oil used. According to the quality criteria of the Turkish Food Codex Communiqué on Oils Called by Plant Name (TGK, 2012), the linoleic acid ratio required in sunflower oils is in the range of 18.7%–74.0%. The myristic acid ratio increased from 0.22% to 1.01% on Day 14, and from 0.48% to 1.58% on Day 28 (*p* < 0.05). There was no significant change in the myristic acid ratios of the SOp + P_1_ and SOp + BHT samples. Myristic acid ratios increased in the SOp + P_4_, SOp + P_5_, SOp + P_6_, SOp + P_11_, SOp + P_13_, SOp + P_16_, and SOr samples depending on the storage time (*p* < 0.05). On Day 14, the highest increase was observed in the samples containing P_5_, P_2_, P_7_, P_12_, P_9_, and P_16_, respectively (*p* < 0.05). At the end of storage (28th day), the highest myristic acid ratios were observed in the SOp + P_10_, SOp + P_5_, and SOr samples. Palmitic acid ratios in the samples showed a fluctuating change instead of a regular change (5.86%–6.56%). Stearic acid content did not show a significant change in P_1_, P_4_, P_14_, P_16_, and BHT‐containing samples depending on the storage period, whereas it showed an increasing trend in the other samples (*p* < 0.05). The oleic acid ratios did not show significant changes in the samples except for P_1_, P_5_, and P_10_ (*p* < 0.05). The oleic acid content decreased in the samples containing P_1_ and P_10_. Linoleic acid, the most dominant fatty acid in sunflower oil, decreased in samples containing P_2_, P_3_, P_5_, P_11_, P_13_, and SOr in parallel with the increase in storage time (*p* < 0.05). The linoleic acid content, which was 58.9% on average at the beginning, decreased to 58.82% on Day 14 and 58.29% on Day 28. The sample with the highest decrease in linoleic acid content was SOr without antioxidants. The linoleic acid content of SOr decreased from 58.96% at the beginning to 51.17% at the end of the storage period (*p* < 0.05). The arachidic acid content did not show a significant change in the samples, except for SOp + P_10_. The total saturated fatty acid (∑SFA) and total unsaturated fatty acid (∑UFA) contents (%) determined in the samples depending on the storage time are given in Table 3. The ∑SFA ratios increased in the samples to which P_2_, P_5_, P_6_, P_8_, P_10_, P_11_, P_13_, and P_16_ were added (*p* < 0.05). The ∑UFA content decreased in the SOr samples during storage, whereas no significant change was detected in the other samples.

**TABLE 3:** Fatty acid compositions of the samples at 60^°^C for three different periods.

Sample	Time	Myristic (C14:0)	Palmitic (C16:0)	Stearic (C18:0)	Oleic (C18:1)	Linoleic (C18:2)	Arachidic (C20:0)	∑SFA	∑UFA
**SOP** + **P_1_**	Initially	0.39±0.04^a^	6.12±0.04^ab^	4.08±0.03^a^	30.96±0.04^b^	57.89±0.06^a^	0.18±0.03^a^	10.77±0.08^ab^	88.85±0.10^a^
	14th day	0.43±0.03^a^	6.16±0.04^b^	4.15±0.04^a^	30.77±0.17^b^	58.22±0.34^ab^	0.20±0.04^a^	10.94±0.07^b^	88.99±0.51^a^
	28th day	0.41±0.04^a^	6.03±0.03^a^	4.05±0.04^a^	30.25±0.18^a^	59.04±0.30^b^	0.19±0.04^a^	10.68±0.01^a^	89.29±0.48^a^
**SOp** + **P_2_**	Initially	—	6.08±0.01^a^	3.92±0.06^ab^	30.29±0.23^a^	59.55±0.48^b^	0.16±0.01^a^	10.16±0.08^a^	89.84±0.71^a^
	14th day	0.95±0.03^b^	6.21±0.04^b^	4.04±0.04^ab^	30.20±0.18^a^	58.22±0.17^a^	0.18±0.01^a^	11.38±0.04^c^	88.42±0.35^a^
	28th day	0.46±0.07^a^	5.99±0.04^a^	4.09±0.03^b^	31.00±0.65^a^	58.27±0.28^a^	0.19±0.03^a^	10.73±0.17^b^	89.27±0.93^a^
**SOp** + **P_3_**	Initially	0.61±0.04^b^	6.20±0.06^a^	3.99±0.04^a^	30.27±0.41^a^	58.69±0.38^b^	0.17±0.03^a^	10.97±0.03^a^	88.96±0.79^a^
	14th day	0.23±0.03^a^	6.20±0.03^a^	4.01±0.04^ab^	30.69±0.21^a^	58.69±0.27^b^	0.18±0.06^a^	10.62±0.16^a^	89.38±0.48^a^
	28th day	—	6.56±0.01^b^	4.15±0.06^b^	31.10±0.4^a^	57.24±0.55^a^	0.25±0.04^a^	10.96±0.11^a^	88.34±0.95^a^
**SOp** + **P_4_**	Initially	0.40±0.01^a^	6.21±0.06^b^	4.01±0.03^a^	30.49±0.28^a^	58.73±0.33^a^	0.16±0.04^a^	10.78±0.11^a^	89.22±0.61^a^
	14th day	0.51±0.04^b^	6.06±0.07^ab^	4.08±0.04^a^	30.57±0.3^a^	58.59±0.23^a^	0.19±0.07^a^	10.84±0.14^a^	89.16±0.52^a^
	28th day	0.58±0.03^b^	5.95±0.06^a^	4.12±0.04^a^	30.27±0.45^a^	58.88±0.06^a^	0.20±0.04^a^	10.85±0.08^a^	89.15±0.51^a^
**SOp** + **P_5_**	Initially	0.66±0.04^a^	6.03±0.03^b^	3.75±0.03^a^	29.43±0.21^a^	59.66±0.14^b^	0.12±0.03^a^	10.56±0.01^a^	89.09±0.35^a^
	14th day	1.01±0.04^b^	6.12±0.01^c^	3.91±0.03^b^	29.91±0.06^b^	58.87±0.14^a^	0.11±0.03^a^	11.15±0.11^b^	88.78±0.20^a^
	28th day	1.50±0.08^c^	5.95±0.01^a^	3.94±0.04^b^	29.85±0.08^ab^	58.26±0.28^a^	0.17±0.03^a^	11.56±0.06^c^	88.11±0.37^a^
**SOp** + **P_6_**	Initially	0.30±0.04^a^	6.09±0.03^b^	3.93±0.03^a^	30.58±0.34^a^	58.93±0.08^a^	0.17±0.01^a^	10.49±0.03^a^	89.51±0.42^a^
	14th day	0.60±0.03^b^	6.10±0.03^b^	4.10±0.04^b^	30.58±0.08^a^	59.39±0.27^a^	0.20±0.01^a^	11.00±0.06^c^	89.97±0.35^a^
	28th day	0.48±0.04^b^	5.94±0.03^a^	4.04±0.01^b^	30.48±0.16^a^	58.84±0.11^a^	0.19±0.04^a^	10.65±0.04^b^	89.32±0.27^a^
**SOp** + **P_7_**	Initially	0.60±0.03^a^	6.12±0.03^b^	3.94±0.04^a^	30.52±0.34^a^	58.66±0.17^ab^	0.16±0.01^a^	10.82±0.03^ab^	89.18±0.51^a^
	14th day	0.81±0.03^b^	5.87±0.06^a^	4.10±0.03^b^	30.45±0.34^a^	58.44±0.25^a^	0.19±0.01^a^	10.97±0.10^b^	88.89±0.59^a^
	28th day	0.56±0.01^a^	5.90±0.03^a^	4.01±0.03^ab^	30.19±0.42^a^	59.16±0.23^b^	0.18±0.03^a^	10.65±0.10^a^	89.35±0.65^a^
**SOp** + **P_8_**	Initially	–	5.92±0.03^a^	3.97±0.03^a^	30.52±0.33^a^	59.43±0.47^a^	0.16±0.01^a^	10.05±0.01^a^	89.95±0.79^a^
	14th day	0.56±0.01^b^	6.09±0.03^b^	4.07±0.03^b^	30.50±0.51^a^	58.50±0.25^a^	0.19±0.04^a^	10.91±0.03^c^	89.00±0.76^a^
	28th day	0.50±0.01^a^	5.92±0.04^a^	4.04±0.03^ab^	30.36±0.25^a^	58.98±0.23^a^	0.20±0.04^a^	10.66±0.04^b^	89.34±0.03^a^
**SOp** + **P_9_**	Initially	0.55±0.03^a^	6.07±0.04^a^	3.97±0.03^ab^	30.55±0.18^a^	58.70±0.13^a^	0.16±0.04^a^	10.75±0.08^a^	89.25±0.31^a^
	14th day	0.72±0.03^b^	6.00±0.06^a^	3.96±0.03^a^	29.93±0.08^a^	59.24±0.20^b^	0.15±0.01^a^	10.83±0.07^a^	89.17±0.28^a^
	28th day	0.51±0.03^a^	6.00±0.04^a^	4.06±0.03^b^	30.44±0.34^a^	58.79±0.17^ab^	0.20±0.01^a^	10.77±0.11^a^	89.23±0.51^a^
**SOp** + **P_10_**	Initially	0.58±0.03^b^	6.08±0.04^b^	3.97±0.03^a^	30.49±0.38^b^	58.61±0.21^a^	0.18±0.01^b^	10.81±0.11^a^	89.10±0.59^a^
	14th day	0.43±0.03^a^	5.95±0.04^a^	4.07±0.01^b^	30.34±0.21^ab^	59.02±0.13^a^	0.19±0.03^b^	10.64±0.06^a^	89.36±0.34^a^
	28th day	1.58±0.03^c^	6.06±0.03^ab^	3.95±0.03^a^	29.43±0.37^a^	58.77±0.16^a^	0.09±0.01^a^	11.68±0.10^b^	88.20±0.52^a^
**SOp** + **P_11_**	Initially	0.35±0.01^a^	5.87±0.01^a^	3.95±0.04^a^	30.40±0.42^a^	59.27±0.16^b^	0.16±0.04^a^	10.33±0.08^a^	89.67±0.58^a^
	14th day	0.53±0.03^b^	5.89±0.03^a^	4.09±0.01^b^	30.53±0.41^a^	58.76±0.23^ab^	0.20±0.03^a^	10.71±0.01^b^	89.29±0.64^a^
	28th day	0.67±0.01^c^	6.12±0.03^b^	4.00±0.06^ab^	30.61±0.49^a^	58.25±0.35^a^	0.18±0.03^a^	10.97±0.04^c^	88.86±0.85^a^
**SOp** + **P_12_**	Initially	0.88±0.04^c^	6.09±0.04^b^	3.82±0.04^a^	29.95±0.24^a^	59.03±0.42^a^	0.12±0.03^a^	10.91±0.16^b^	88.98±0.66^a^
	14th day	0.73±0.04^b^	6.17±0.01^b^	4.03±0.03^b^	30.20±0.31^a^	58.70±0.17^a^	0.17±0.04^a^	11.10±0.04^b^	88.90±0.48^a^
	28th day	0.14±0.01^a^	5.90±0.04^a^	4.05±0.03^b^	30.23±0.40^a^	59.49±0.14^a^	0.19±0.03^a^	10.28±0.08^a^	89.72±0.54^a^
**SOp** + **P_13_**	Initially	0.42±0.06^a^	5.85±0.04^a^	3.94±0.01^a^	30.62±0.17^a^	58.99±0.14^b^	0.18±0.01^a^	10.39±0.10^a^	89.61±0.03^a^
	14th day	0.54±0.04^a^	5.99±0.03^b^	3.99±0.03^a^	30.21±0.3^a^	59.11±0.01^b^	0.16±0.03^a^	10.68±0.01^b^	89.32±0.31^a^
	28th day	0.57±0.01^b^	6.04±0.03^b^	4.11±0.03^b^	30.45±0.04^a^	58.61±0.08^a^	0.19±0.03^a^	10.91±0.01^c^	89.06±0.13^a^
**SOp** + **P_14_**	Initially	0.51±0.03^ab^	6.21±0.04^b^	4.01±0.04^a^	30.48±0.2^a^	58.62±0.28^a^	0.17±0.03^a^	10.90±0.14^a^	89.10±0.48^a^
	14th day	0.45±0.03^a^	5.92±0.04^a^	4.04±0.04^a^	30.39±0.45^a^	59.01±0.42^a^	0.19±0.01^a^	10.60±0.13^a^	89.40±0.88^a^
	28th day	0.57±0.04^b^	5.94±0.04^a^	4.06±0.04^a^	30.07±0.30^a^	59.17±0.34^a^	0.19±0.01^a^	10.76±0.06^a^	89.24±0.64^a^
**SOp** + **P_15_**	Initially	0.70±0.04^b^	6.21±0.04^a^	3.99±0.03^a^	30.14±0.20^a^	58.80±0.27^a^	0.16±0.01^a^	11.06±0.07^a^	88.94±0.47^a^
	14th day	0.58±0.04^ab^	6.17±0.07^a^	4.04±0.03^ab^	30.31±0.37^a^	58.70±0.23^a^	0.17±0.03^a^	10.96±0.08^a^	89.01±0.59^a^
	28th day	0.55±0.03^a^	6.18±0.07^a^	4.13±0.04^b^	30.43±0.08^a^	58.48±0.4^a^	0.20±0.03^a^	11.06±0.17^a^	88.91±0.48^a^
**SOp** + **P_16_**	Initially	0.40±0.03^a^	6.15±0.06^a^	4.05±0.04^a^	30.37±0.34^a^	58.84±0.11^a^	0.19±0.06^a^	10.79±0.01^a^	89.21±0.45^a^
	14th day	0.71±0.06^c^	6.36±0.03^b^	4.04±0.04^a^	30.00±0.14^a^	58.72±0.24^a^	0.17±0.01^a^	11.28±0.06^c^	88.72±0.38^a^
	28th day	0.56±0.04^b^	6.34±0.04^b^	4.00±0.04^a^	30.33±0.23^a^	58.57±0.58^a^	0.17±0.01^a^	11.07±0.03^b^	88.90±0.81^a^
**SOp**	Initially	0.81±0.03^c^	6.03±0.04^a^	3.95±0.04^a^	30.00±0.64^a^	58.92±0.23^a^	0.16±0.01^a^	10.95±0.13^b^	88.92±0.41^a^
	14th day	0.40±0.06^a^	5.92±0.04^a^	4.04±0.04^ab^	30.25±0.38^a^	59.20±0.30^a^	0.19±0.03^a^	10.55±0.00^a^	89.45±0.68^a^
	28th day	0.58±0.04^b^	6.22±0.04^b^	4.11±0.04^b^	30.46±0.21^a^	58.32±0.41^a^	0.19±0.04^a^	11.10±0.08^b^	88.78±0.62^a^
**SOr**	Initially	0.33±0.06^a^	6.27±0.03^b^	4.05±0.03^b^	30.05±0.34^a^	58.96±0.14^b^	0.17±0.03^a^	10.82±0.14^b^	89.01±0.48^b^
	14th day	0.22±0.03^a^	6.09±0.04^a^	4.02±0.03^ab^	30.30±0.42^a^	59.08±0.28^b^	0.17±0.01^a^	10.50±0.08^a^	89.38±0.71^b^
	28th day	1.06±0.03^b^	6.26±0.03^b^	3.95±0.03^a^	29.73±0.21^a^	51.17±0.42^a^	0.19±0^a^	11.46±0.03^c^	80.90±0.64^a^
**SOp** + **BHT**	Initially	0.50±0.04^a^	6.16±0.04^b^	4.00±0.04^a^	30.36±0.25^a^	58.82±0.16^a^	0.16±0.01^a^	10.82±0.14^ab^	89.18±0.41^a^
	14th day	0.47±0.06^a^	5.86±0.01^a^	3.98±0.04^a^	30.26±0.23^a^	59.24±0.31^a^	0.19±0.03^a^	10.50±0.03^a^	89.50±0.54^a^
	28th day	0.60±0.04^a^	6.13±0.06^b^	4.05±0.04^a^	29.76±0.23^a^	59.30±0.34^a^	0.17±0.01^a^	10.95±0.13^b^	89.06±0.57^a^

*Note*: Different lowercase letters given as exponents in the same column of a sample indicate that the difference between the means is significant (*p* < 0.05). SOr: refined sunflower oil, SOp: sunflower oil stripped of antioxidants, SOp + BHT: pure sunflower oil with 200 ppm BHT, SOp + P_1_: SOp with 1000 ppm P_1_ added, SOp + P_2_: SOp with 1000 ppm P_2_ added, SOp + P_3_: SOp with 1000 ppm P_3_ added, SOp + P_4_: SOp with 1000 ppm P_4_ added, SOp + P_5_: SOp with 1000 ppm P_5_ added, SOp + P_6_: SOp with 1000 ppm P_6_ added, SOp + P_7_: SOp with 1000 ppm P_7_ added, SOp + P_8_: SOp with 1000 ppm P_8_ added, SOp + P_9_: SOp with 1000 ppm P_9_ added, SOp + P_10_: SOp with 1000 ppm P_10_ added, SOp + P_11_: SOp with 1000 ppm P_11_ added, SOp + P_12_: SOp with 1000 ppm P_12_ added, SOp + P_13_: SOp with 1000 ppm P_13_ added, SOp + P_14_: SOp with 1000 ppm P_14_ added, SOp + P_15_: SOp with 1000 ppm P_15_ added, SOp + P_16_: SOp with 1000 ppm P_16_ added.

## 4 CONCLUSIONS

This study assessed the TPC and antioxidant activity of propolis collected from 16 different regions of Türkiye. It also examined into the effect of these propolis methanol extracts on the oxidative stability of sunflower oil stored at 60°C. Thirteen propolis extracts (ranging from 28.1% to 92.5%) exhibited greater DPPH radical scavenging activity than BHT (24.5%). The ABTS values of propolis extracts were between 193.8 and 1369.6 µmol trolox/g, whereas that of BHT was 224 µmol trolox/g. The TPC ranged from 5333.3 to 36,966.7 mg GAE/100 g. A significant positive correlation was observed between the ABTS, TPC, and DPPH values. Most of the propolis extracts added to sunflower oil at 1000 ppm concentration, especially P_11_, P_15_, P_14_, P_7_, P_16_, P_5_, and P_12_, showed better antioxidant effects than BHT added at 200 ppm concentration. In general, the sunflower oils to which these extracts were added showed lower levels of PV, p‐AV, TOTOX, K_232_, and K_270_. Consequently, the use of propolis extracts as natural antioxidants could lead to the development of healthier and more stable edible oils, reducing the dependence on synthetic antioxidants. The rich chemical composition and valuable properties of propolis offer great potential for application in the food industry as a natural and safe preservative with high antioxidant activity, and as a means of enhancing the nutritional value of foods. By reducing lipid oxidation, propolis can prevent undesirable physical, chemical, and organoleptic changes in foods, thus preserving the quality and extending the shelf life of food products of plant and animal origin. However, further studies should be carried out to determine the individual phenolic content and volatile components of prolises and to determine which components have high antioxidant effects. More work should be done with propolis, not only for fats, but also to improve the quality, nutritional value, and shelf life of fat‐containing foods.

## AUTHOR CONTRIBUTIONS

**Ayhan Baştürk**: Investigation; writing—original draft; writing—review and editing; project administration; supervision; visualization; conceptualization. **Berfin Yavaş**: Investigation; writing—original draft; formal analysis; methodology; writing—review and editing; resources.

## CONFLICT OF INTEREST STATEMENT

The authors declare that they have no known conflicts of interest or personal relationships that could have appeared to influence the work reported in this paper.

## Supporting information



Table S1 Correlation between DPPH, ABTS, and TPC.

Table S2 Free fatty acid (FFA) content (% oleic acid) of sunflower oil samples with different propolis extracts and BHT, measured at various storage periods at 60°C.

## Data Availability

Data will be made available on request.
