# Supplementary material for: Improving sunflower oil stability with propolis: A study on antioxidative effects of Turkish propolis during accelerated oxidation
Source: J Food Sci. 2024 Oct 30;89(12):8910–29. doi: 10.1111/1750-3841.17482 (PMC11673529; doi:10.1111/1750-3841.17482)
Supplement: Supplementary file 1 — Table S1 Correlation between DPPH, ABTS, and TPC. [file JFDS-89-8910-s001.docx]

**Table S1**

Correlation between DPPH, ABTS and TPC

|  | | DPPH | ABTS | TPC |
| --- | --- | --- | --- | --- |
| DPPH | Pearson Correlation | 1 | 0.796^**^ | 0.936^**^ |
|  | Sig. (2-tailed) |  | 0.000 | 0.000 |
|  | N | 34 | 34 | 32 |
|  |  |  |  |  |
| ABTS | Pearson Correlation | 0.796^**^ | 1 | 0.786^**^ |
|  | Sig. (2-tailed) | 0.000 |  | 0.000 |
|  | N | 34 | 34 | 32 |
|  |  |  |  |  |
| TPC | Pearson Correlation | 0.936^**^ | 0.786^**^ | 1 |
|  | Sig. (2-tailed) | 0.000 | 0.000 |  |
|  | N | 32 | 32 | 32 |

**. Correlation is significant at the 0.01 level (2-tailed).
