# Supplementary material for: Improving sunflower oil stability with propolis: A study on antioxidative effects of Turkish propolis during accelerated oxidation
Source: J Food Sci. 2024 Oct 30;89(12):8910–29. doi: 10.1111/1750-3841.17482 (PMC11673529; doi:10.1111/1750-3841.17482)
Supplement: Supplementary file 2 — Table S2 Free fatty acid (FFA) content (% oleic acid) of sunflower oil samples with different propolis extracts and BHT, measured at various storage periods at 60°C. [file JFDS-89-8910-s002.docx]

**Table S2**

Free fatty acid (FFA) content (% oleic acid) of sunflower oil samples with different propolis extracts and BHT, measured at various storage periods at 60°C.

| **Sample** | **Initially** | | **7th day** | | **14th day** | | **21st day** | | **28th day** | |
| --- | --- | --- | --- | --- | --- | --- | --- | --- | --- | --- |
| SOp+P_1_ | 0.115 | ±0.012^bcA^ | 0.175 | ±0.018^defB^ | 0.245 | ±0.029^eC^ | 0.156 | ±0.009^efAB^ | 0.146 | ±0.017^cdeAB^ |
| SOp+P_2_ | 0.127 | ±0.011^cdeA^ | 0.193 | ±0.015^fB^ | 0.189 | ±0.010^cdB^ | 0.135 | ±0.011^cdeA^ | 0.112 | ±0.011^bcA^ |
| SOp+P_3_ | 0.209 | ±0.023^ıB^ | 0.122 | ±0.015^bcA^ | 0.142 | ±0.020^bA^ | 0.110 | ±0.014^bcdA^ | 0.113 | ±0.015^bcA^ |
| SOp+P_4_ | 0.165 | ±0.014^fghB^ | 0.188 | ±0.012^efB^ | 0.187 | ±0.013^cdB^ | 0.127 | ±0.010^bcdeA^ | 0.158 | ±0.007^eB^ |
| SOp+P_5_ | 0.151 | ±0.015^defghA^ | 0.165 | ±0.013^defA^ | 0.170 | ±0.015^bcdA^ | 0.135 | ±0.013^deA^ | 0.157 | ±0.010^eA^ |
| SOp+P_6_ | 0.126 | ±0.013^cdeA^ | 0.143 | ±0.012^cdAB^ | 0.169 | ±0.008^bcdB^ | 0.124 | ±0.012^bcdeA^ | 0.120 | ±0.014^bcdA^ |
| SOp+P_7_ | 0.137 | ±0.007^cdefgAB^ | 0.164 | ±0.030^defAB^ | 0.173 | ±0.012^bcdB^ | 0.123 | ±0.020^bcdeA^ | 0.143 | ±0.014^cdeAB^ |
| SOp+P_8_ | 0.156 | ±0.019^efghAB^ | 0.176 | ±0.013^defB^ | 0.184 | ±0.018^cdB^ | 0.146 | ±0.012^efAB^ | 0.133 | ±0.013^cdeA^ |
| SOp+P_9_ | 0.130 | ±0.015^cdefA^ | 0.173 | ±0.013^defB^ | 0.183 | ±0.020^cdB^ | 0.123 | ±0.015^bcdeA^ | 0.157 | ±0.010^eAB^ |
| SOp+P_10_ | 0.120 | ±0.013^bcdA^ | 0.145 | ±0.011^cdA^ | 0.158 | ±0.010^bcA^ | 0.123 | ±0.019^bcdeA^ | 0.135 | ±0.014^cdeA^ |
| SOp+P_11_ | 0.133 | ±0.015^cdefAB^ | 0.140 | ±0.015^cdAB^ | 0.162 | ±0.018^bcdBC^ | 0.101 | ±0.016^bA^ | 0.203 | ±0.026^fC^ |
| SOp+P_12_ | 0.136 | ±0.012^cdefgABC^ | 0.156 | ±0.010^cdefBC^ | 0.166 | ±0.010^bcdC^ | 0.111 | ±0.014^bcdA^ | 0.130 | ±0.007^cdeAB^ |
| SOp+P_13_ | 0.114 | ±0.015^bcAB^ | 0.154 | ±0.016^cdeBC^ | 0.173 | ±0.017^bcdC^ | 0.101 | ±0.014^bcA^ | 0.112 | ±0.014^bcA^ |
| SOp+P_14_ | 0.169 | ±0.015^ghAB^ | 0.185 | ±0.013^efB^ | 0.198 | ±0.014^dB^ | 0.134 | ±0.011^bcdeA^ | 0.142 | ±0.016^cdeA^ |
| SOp+P_15_ | 0.143 | ±0.013^cdefgAB^ | 0.164 | ±0.015^defB^ | 0.178 | ±0.011^bcdB^ | 0.124 | ±0.018^bcdeA^ | 0.148 | ±0.012^cdeAB^ |
| SOp+P_16_ | 0.184 | ±0.016^hıA^ | 0.191 | ±0.028^efA^ | 0.194 | ±0.025^cdA^ | 0.169 | ±0.008^fA^ | 0.152 | ±0.017^deA^ |
| SOp | 0.091 | ±0.012^abAB^ | 0.097 | ±0.005^abB^ | 0.108 | ±0.012^aB^ | 0.067 | ±0.015^aA^ | 0.090 | ±0.001^bAB^ |
| SOr | 0.165 | ±0.012^fghA^ | 0.177 | ±0.012^defA^ | 0.173 | ±0.013^bcdA^ | 0.146 | ±0.008^efA^ | 0.236 | ±0.025^gB^ |
| SOp+BHT | 0.080 | ±0.011^aBC^ | 0.086 | ±0.008^aC^ | 0.099 | ±0.004^aC^ | 0.064 | ±0.008^aAB^ | 0.056 | ±0.006^aA^ |

Values represent the percentage of oleic acid, indicating the hydrolytic degradation of triglycerides.

Different lowercase letters given as exponents in the same column indicate a significant difference between the means (p<0.05).

Different capital letters given as exponents in the same row indicate a significant difference between the averages depending on the duration (p<0.05).
